# Circulating retinol binding protein 4 in critically ill patients before specific treatment: prognostic impact and correlation with organ function, metabolism and inflammation

**DOI:** 10.1186/cc9285

**Published:** 2010-10-08

**Authors:** Alexander Koch, Ralf Weiskirchen, Edouard Sanson, Henning W Zimmermann, Sebastian Voigt, Hanna Dückers, Christian Trautwein, Frank Tacke

**Affiliations:** 1 Department of Medicine III, RWTH-University Hospital Aachen, Pauwelsstrasse 30, 52074 Aachen, Germany; 2 Institute of Clinical Chemistry and Pathobiochemistry, RWTH-University Hospital Aachen, Pauwelsstrasse 30, 52074 Aachen, Germany

## Abstract

**Introduction:**

Hyperglycemia and insulin resistance are well-known features of critical illness and impact the mortality rate, especially in sepsis. Retinol binding protein 4 (RBP4) promotes insulin resistance in mice and is systemically elevated in patients with obesity and type 2 diabetes. We investigated the potential role of RBP4 in critically ill patients.

**Methods:**

We conducted a prospective single-center study of serum RBP4 concentrations in critically ill patients. One hundred twenty-three patients (85 with sepsis, 38 without sepsis) were studied at admission to a medical intensive care unit (ICU) before initiation of specific intensive care treatment measures and compared to 42 healthy nondiabetic controls. Clinical data, various laboratory parameters and metabolic and endocrine functions were assessed. Patients were followed for approximately 3 years.

**Results:**

Serum RBP4 was significantly reduced in ICU patients, independently of sepsis, as compared to healthy controls (*P *< 0.001). Patients with liver cirrhosis as the primary underlying diagnosis for ICU admission had significantly lower RBP4 levels as compared with other ICU patients. Accordingly, in all ICU patients, serum RBP4 closely correlated with liver function and increased with renal failure. No significant differences of serum RBP4 concentrations in septic patients with pulmonary or other origins of sepsis or nonseptic patients could be revealed. Acute phase proteins were inversely correlated with RBP4 in sepsis patients. RBP4 did not differ between patients with or without obesity or preexisting diabetes. However, serum RBP4 levels correlated with endogenous insulin secretion (C-peptide) and insulin resistance (HOMA index). Low serum RBP4 upon admission was an adverse predictor of short-term survival in the ICU, but was not associated with overall survival during long-term follow-up.

**Conclusions:**

Serum RBP4 concentrations are significantly reduced in critically ill patients. The strong associations with hepatic and renal function, insulin resistance and acute mortality collectively suggest a role of RBP4 in the pathogenesis of critical illness, possibly as a negative acute phase reactant, and allow a proposition as a potential novel biomarker for ICU patients.

## Introduction

Hyperglycemia, glucose intolerance and insulin resistance are common features of critically ill patients, especially in patients with sepsis or septic shock and even in those without a history of diabetes [1-3]. Maintenance of normoglycemia (blood glucose levels of 110 mg/dL) with intensive insulin therapy has been shown to improve survival and reduce morbidity in patients with prolonged critically illness after cardiac surgery [4], whereas its beneficial effect on the outcome of patients in medical intensive care units (ICU) is controversial [5,6]. In patients with obesity, metabolic syndrome and type 2 diabetes, several adipocytokines have been identified that mediate agonistic and antagonistic effects on insulin resistance [7,8]. In these patients, chronic inflammatory conditions apparently promote adipocytic secretion of mediators of insulin resistance into the circulation, thereby providing a link between adipocytokines, inflammation and systemic insulin resistance [7,8].

Retinol binding protein 4 (RBP4), a 21-kDa protein synthesized in the liver and adipose tissue, has recently been described as an adipokine involved in the development of insulin resistance in mice and humans [9,10]. In the past, RBP4 was solely recognized for its role as the specific transport protein for vitamin A (retinol) in the circulation, whose function is to deliver hydrophobic retinol from the liver to target tissues [11]. In animal models of insulin resistance, the expression of RBP4 was strongly induced in adipose tissue, and systemic release of RBP4 appeared to be a crucial signal for the development of systemic insulin resistance [10,12]. These results have been translated into the pathogenesis of insulin resistance in humans. Serum RBP4 correlated positively with the presence of insulin resistance in individuals with obesity, impaired glucose tolerance or type 2 diabetes [13,14]. Elevated serum RBP4 concentrations were an independent predictive biomarker at early stages of insulin resistance identifying individuals at risk of developing diabetes and were even found in healthy individuals with a strong family history of type 2 diabetes [13,14].

Recently, low levels of serum RBP4 have been reported in critical ill patients with sepsis of pulmonary origin compared to nonseptic patients [15]. However, it remained unclear whether this observation was related to sepsis or whether it could be extrapolated to all critically ill patients, because this study analyzed only patients with a respiratory disease as the main reason for admission to the ICU. Moreover, the pathogenic and/or diagnostic relevance of RBP4 in ICU patients is presently not known [15]. Various essential therapeutic interventions in the initial phase of intensive care treatment, e.g., fluid challenge, insulin therapy, nutritional support and renal replacement therapies, potentially influence concentrations of biomarkers. At present, there is a lack of studies investigating the regulation of adipocytokines in critical illness at the point of admission before any substantial therapeutic interference.

Our study investigated serum RBP4 concentrations in a large cohort of "untouched," treatment-naive critically ill patients (septic and nonseptic patients) from a medical ICU at the moment of admission to the ICU. We aimed at understanding the potential involvement of RBP4 in the pathogenesis of insulin resistance in critical illness, its regulation in severe systemic inflammation and its potential clinical use as a biomarker in ICU patients. We demonstrate that serum RBP4 levels were significantly reduced in ICU patients as compared to controls, independent of etiology of critical illness or origin of sepsis (pulmonary versus nonpulmonary), and they were closely related to liver and kidney function. The dysregulation of serum RBP4 in ICU patients was not associated with preexisting diabetes or obesity, but might contribute to the insulin-resistant state of the critically ill patients. High RBP4 levels were indicative of a beneficial short-term course of disease, but did not predict the overall survival of the patients in a 3-year follow-up period.

## Materials and methods

### Patients and controls

From the medical ICU at the University Hospital Aachen, Germany, a tertiary care university hospital, patients were prospectively enrolled at the time of ICU admission. Patients who were expected to stay < 72 hours at the ICU (e.g., postinterventional observation, acute intoxication) were not asked to participate in this study [16]. Written informed consent was obtained from each participant or his or her spouse, and the study was approved by the local ethics committee (ethics committee of the University Hospital Aachen, RWTH-University, Aachen, Germany, reference number EK 150/06). Patient data, blood samples and clinical information were collected prospectively. Owing to lack of sufficient material, 123 of initially 170 consecutively recruited patients were included in the current study. Median duration of stay at the ICU was 8 days (range, 1-137 days), and median duration of stay at the hospital was 26.5 days (range, 2-151 days). The clinical course of the patients after discharge was followed by contacting the patients and/or their primary care physician up to 3 years after entry into this study (median observation time, 591 days; range, 29-884 days). Patients were categorized as having severe sepsis (acute organ dysfunction secondary to infection) and septic shock (severe sepsis plus hypotension not reversed with fluid resuscitation), hereafter called *sepsis*, according to the definitions in current guidelines [17], or as being *nonsepsis. Sepsis *patients were subdivided into cohorts of *sepsis of pulmonary *and *nonpulmonary origin *on the basis of the clinical and radiological classification performed upon admission.

Forty-two healthy, nondiabetic blood donors (30 male, 12 female; median age, 53 years; range, 24-68 years) who had normal blood counts, normal liver enzymes and normal C-reactive protein levels served as controls.

### Comparative and laboratory variables

The ICU patients were compared by age, sex and severity of illness using the score on the Acute Physiology and Chronic Health Evaluation (APACHE) II obtained at admission and the simplified acute physiology score (SAPS) 2 [16]. Laboratory parameters were routinely assessed at admission, recorded and analyzed.

### Quantification of RBP4 serum levels and other adipocytokines

Peripheral venous blood samples were obtained at admission before therapeutic intervention, immediately placed on ice, centrifuged and stored at -80°C. All measurements were performed in a blinded fashion by the same investigator. RBP4 serum concentrations were measured using an enzyme-linked immunosorbent assay according to the manufacturer's instructions (Immundiagnostik AG, Bensheim, Germany) [18]. Serum resistin, ghrelin, adiponectin and C-peptide were measured as described previously [19-21]. Insulin sensitivity was assessed by the homeostasis model assessment (HOMA) index using the HOMA Calculator V. 2.2.2 from the University of Oxford, UK [20,22].

### Statistical analysis

Analyses were performed with SPSS 12.0 software (SPSS, Munich, Germany). Owing to skewed distributions of most variables, the median and range are given. Differences between two groups were assessed by the Mann-Whitney *U *test or between more than two groups by Kruskal-Wallis analysis of variance and the Mann-Whitney *U *test for post hoc analysis [18]. Comparisons between subgroups are illustrated with boxplot graphics, where the black bold line indicates the median per group, the box represents 50% of the values and horizontal lines show minimum and maximum values of the calculated nonoutlier values; asterisks and open circles indicate outlier values. The correlations between variables were analyzed using the Spearman correlation tests. Values of *P *< 0.05 were considered statistically significant. The prognostic value of the variables was tested by univariate and multivariate analyses in the Cox regression model. After significant results from the uni- and multivariate Cox regression analyses, Kaplan-Meier curves and log-rank test calculations were performed subsequently for different cutoff values for RBP4 (9, 10, 11, 12, 13, 14 and 15 mg/L). The threshold of 12 mg/L RBP4 yielded the highest log-rank values. Kaplan-Meier curves were plotted to display the impact on survival [23].

## Results

### RBP4 serum levels are significantly reduced in ICU patients compared with healthy controls, independent of the presence of sepsis

**Figure 1:**
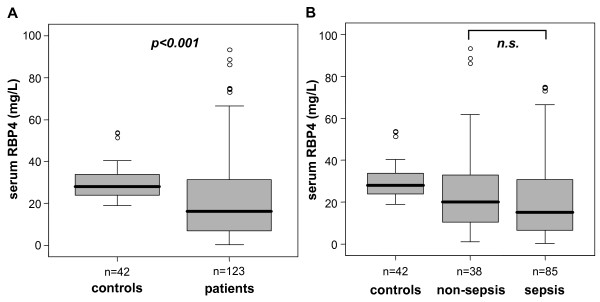
**Serum retinol binding protein 4 (RBP4) concentrations in critically ill patients**. **(a) **Serum RBP4 levels are significantly (*P *< 0.001, Mann-Whitney *U *test) reduced in critically ill patients (*n *= 123) as compared to healthy controls (*n *= 42). **(b) **No significant differences were detected between ICU patients with sepsis and nonseptic etiology of critical illness. Boxplots are displayed, where the bold line indicates the median per group, the box represents 50% of the values, and horizontal lines show minimum and maximum values of the calculated nonoutlier values; asterisks and open circles indicate outlier values.

The median serum concentration of RBP4 in healthy controls was 28.1 mg/L (range, 18.8-255.6 mg/L, 90% interval, 20.0-53.8 mg/L) as anticipated from previous studies using the same assay [13,18]. In our cohort of nondiabetic healthy controls, serum RBP4 did not correlate with the body mass index (BMI), and there was no difference between male and female volunteers. Critically ill ICU patients had significantly reduced serum RBP4 concentrations compared with healthy controls (median, 16.1 vs. 28.1 mg/L, *P *< 0.001; Figure 1a, Table 1). Again, male and female patients did not differ in serum RBP4.

**Table 1:** Comparison between ICU patients and healthy controls

Parameter	Controls	ICU patients	*P *value
Number	42	123	
Sex (male/female)	30/12	81/42	n.s.
Age median (range) (yr)	53 (24-68)	64 (18-81)	< 0.001
BMI median (range) (m^2^/kg)	27.0 (22.0-57.0)	26.1 (15.3-59.5)	n.s.
RBP4 median (range) (mg/L)	28.1 (18.8-255.6)	16.1 (0.3-147.6)	< 0.001

As inflammatory disorders could considerably impact serum RBP4 concentrations, we next compared patients with sepsis (*n *= 85) with patients without sepsis (*n *= 38). In the sepsis group, the focus of infection was either pulmonary (*n *= 45), abdominal (*n *= 19) or other (*n *= 21, e.g., catheter-associated, urogenital or unknown). In the nonsepsis group, the etiology of critical illness was decompensated liver cirrhosis (*n *= 11), cardiopulmonary (*n *= 16) or other diseases (*n *= 11). Interestingly, serum RBP4 did not differ significantly between patients with sepsis (median 15.2 mg/L) and without sepsis (median 20.0 mg/L; Figure 1b). With respect to several other clinical parameters (APACHE II score, mechanical ventilation, vasopressor demand), patients with or without sepsis did not differ, but patients with sepsis had a higher ICU and overall mortality as well as significantly longer ventilation duration (Table 2).

**Table 2:** Baseline patient characteristics and RBP4 serum concentrations

Parameter	All patients	Sepsis	Nonsepsis
Number	123	85	38
Sex (male/female)	81/42	56/29	25/13
Age median (range) (yr)	64 (18-81)	64 (21-81)	60 (18-79)
APACHE-II score median (range)	14 (0-31)	14 (0-31)	14 (0-31)
SAPS2 score median (range)	42 (0-80)	42 (0-79)	42 (13-80)
ICU days median (range)	8 (1-137)	10 (1-137)	5.5** (1-45)
Hospital days median (range)	26 (2-151)	30 (3-151)	14** (2-65)
Death during ICU n (%)	33 (27%)	25 (29%)	8 (21%)
Death during follow-up n (%)	59 (48%)	41 (48%)	18 (47%)
30-day mortality (%)	26.0	25.9	26.3
60-day mortality (%)	34.4	35.7	31.6
90-day mortality (%)	40.2	42.7	34.3
180-day mortality (%)	43.5	46.9	35.3
1-year mortality (%)	52.4	54.1	48.4
Mechanical ventilation n (%)	78 (63%)	53 (62%)	25 (66%)
Ventilation time median (range) (h)	38 (0-2966)	80 (0-2966)	24** (0-755)
pre-existing diabetes n (%)	42 (34%)	27 (32%)	15 (40%)
BMI median (range) (m^2^/kg)	26.1 (15.3-59.5)	26.2 (15.3-59.5)	25.4 (19.0-53.3)
RBP4 median (range) (mg/L)	16.1 (0.3-147.6)	15.2 (0.3-147.6)	20.0 (1.0-118.6)

APACHE, Acute Physiology and Chronic Health Evaluation; SAPS, simplified acute physiology score; ICU, intensive care unit; BMI, body mass index

Significant differences between sepsis and nonsepsis patients are marked by *P < 0.05 or **P < 0.001.

### RBP4 serum levels are associated with liver and renal function and are inversely correlated with inflammatory biomarkers

**Figure 2:**
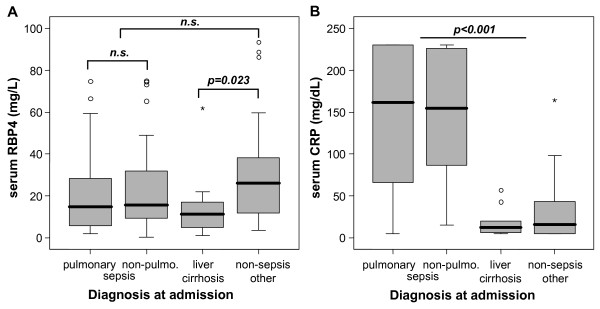
**Impact of primary diagnosis on serum RBP4 concentrations at admission to the medical intensive care unit (ICU)**. **(a) **Among critically ill patients, serum RBP4 levels do not differ between patients with sepsis of pulmonary origin as compared to sepsis of nonpulmonary (non-pulmo.) origin. Lowest RBP4 levels are found in patients with decompensated liver cirrhosis. *P *values (Mann-Whitney *U *test) are given in the figure. **(b) **No significant differences are detected for C-reactive protein (CRP) concentrations in ICU patients with either sepsis of pulmonary or nonpulmonary origin, but both sepsis groups show higher values than the nonsepsis groups. Boxplots are displayed, where the bold line indicates the median per group, the box represents 50% of the values, and horizontal lines show minimum and maximum values of the calculated nonoutlier values; asterisks and open circles indicate outlier values.

A recent study reported reduced RBP4 serum levels in patients with sepsis of pulmonary origin [15]. To investigate the regulation of RBP4 in patients with different etiologies of sepsis and septic shock, we performed extensive subgroup analyses. However, there was no difference in RBP4 serum concentrations between patients with sepsis of pulmonary or nonpulmonary origin (Figure 2a). Furthermore, patients with pulmonary and nonpulmonary sepsis did not differ in baseline clinical parameters or in inflammatory markers such as CRP (Figure 2b, and data not shown).

**Figure 3:**
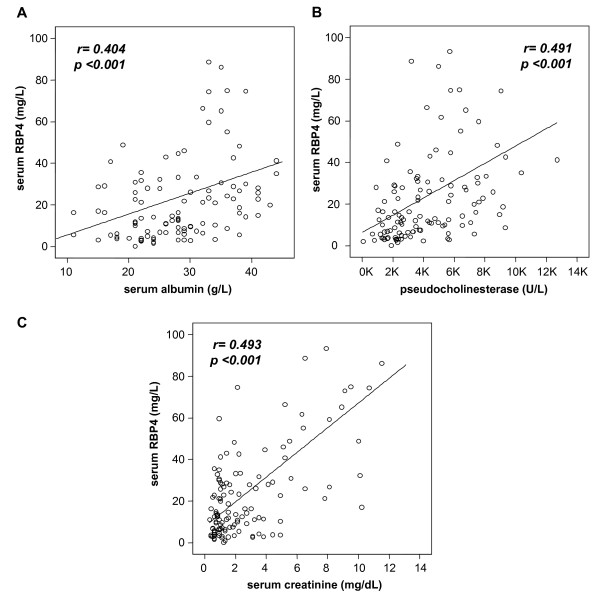
**Impact of organ dysfunction on serum RBP4 in critically ill patients**. **(a **and **b) **Parameters indicating the hepatic biosynthetic capacity, such as serum albumin concentrations or the pseudocholinesterase acitvity, are positively correlated with serum RBP4 concentrations. **(c) **Serum RBP4 is elevated in ICU patients with renal failure, as demonstrated exemplarily by the correlation with serum creatinine. Spearman rank correlation test, correlation coefficient *r *and *P *values are given.

Interestingly, ICU patients with liver cirrhosis displayed the lowest RBP4 serum levels among all subgroups (Figure 2a). Liver function could be identified as a strong predictor of serum RBP4, as RBP4 levels directly correlated with parameters indicating the liver's biosynthetic capacity, namely, albumin (*r *= 0.404, *P *< 0.001; Figure 3a), total protein (*r *= 0.272, *P *= 0.003), pseudocholinesterase activity (*r *= 0.491, *P *< 0.001; Figure 3b), IGF-1 concentrations (*r *= 0.403, *P *< 0.001), prothrombin time (*r *= 0.524, *P *< 0.001) and inversely with bilirubin (e.g., conjugated bilirubin; *r *= -0.434, *P *< 0.001). Of note, RBP4 serum concentrations remained significantly (*P *< 0.001, Mann-Whitney *U *test) elevated in controls (*n *= 42, median 28.1; range, 18.8-255.6 mg/L) compared to ICU patients without cirrhosis (*n *= 112, median 16.5, range 0.3-147.6 mg/L).

Serum RBP4 concentrations were also correlated with markers of renal failure, specifically increasing creatinine (*r *= 0.493, *P *< 0.001; Figure 3c), urea (*r *= 0.389, *P *< 0.001), cystatin C (*r *= 0.381, *P *< 0.001) and decreasing glomerular filtration rate (GFR; *r *= -0.526, *P *< 0.001).

Although there was no significant difference between septic and nonseptic patients, systemic inflammatory markers were associated with serum RBP4 concentrations. Classical biomarkers of systemic inflammatory responses were inversely associated with serum RBP4, such as C-reactive protein (*r *= -0.204, *P *= 0.025) or interleukin-6 levels (*r *= -0.272, *P *= 0.019). RBP4 thereby displayed characteristics of a negative acute phase reactant [24]. However, this association was observed only in sepsis patients.

### Preexisting diabetes or obesity do not significantly impact RBP4 serum levels in ICU patients, but serum RBP4 correlates with insulin secretion and resistance

**Figure 4:**
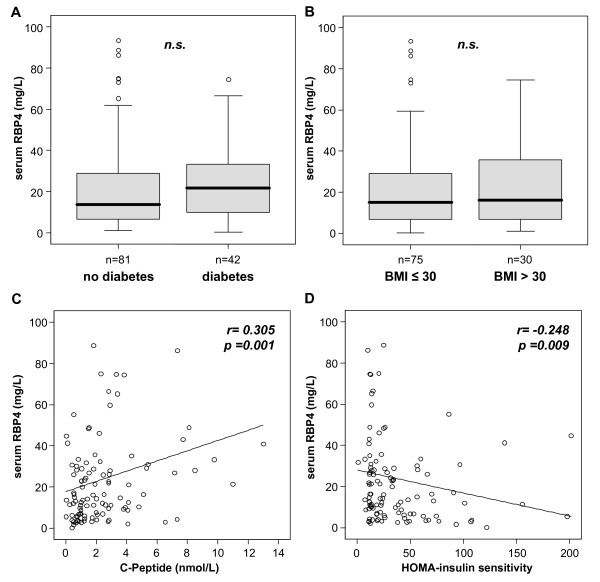
**Association of serum RBP4 with diabetes, obesity and insulin resistance in critically ill patients**. **(a **and **b) **Serum RBP4 levels do not differ between patients with or without preexisting type 2 diabetes on admittance to the ICU **(a) **or with preexisting obesity as defined by a body mass index > 30 kg/m^2 ^**(b)**. Boxplots are displayed, where the bold line indicates the median per group, the box represents 50% of the values, and horizontal lines show minimum and maximum values of the calculated nonoutlier values; asterisks and open circles indicate outlier values. **(c **and **d) **Serum RBP4 concentrations in ICU patients are correlated with C-peptide concentrations and inversely with the insulin sensitivity as calculated by the homeostasis model assessment (HOMA) index. Spearman rank correlation test, correlation coefficient *r *and *P *values are given.

Initial studies linked elevated serum RBP4 to overt or impending insulin resistance in lean, obese and type 2 diabetic subjects [13]. To understand potential pathophysiological consequences of reduced RBP4 levels in ICU patients, we analyzed associations between serum RBP4 and insulin resistance as well as diabetes. Patients with a preexisting type 2 diabetes (*n *= 42) did not differ from patients without diabetes (*n *= 81; Figure 4a) in serum RBP4 concentrations. Consequently, serum RBP4 was not correlated to the glycosylated hemoglobin (HbA1c) levels in ICU patients either. Furthermore, serum RBP4 did not correlate with the patients' BMI (data not shown), and obese patients (defined as BMI > 30, *n *= 30) did not show higher serum RBP4 than patients without severe obesity (BMI ≤ 30, *n *= 75; Figure 4b).

Although serum RBP4 did not correlate with serum glucose on admission to the ICU, serum RBP4 correlated with the C-peptide (*r *= 0.305, *P *= 0.001; Figure 4c), with the insulin resistance as calculated by the HOMA index (HOMA-IR; *r *= 0.248, *P *= 0.009; HOMA-S, *r *= -0.248, *P *= 0.009; Figure 4d). This association of serum RBP4 to endogenous insulin secretion and insulin resistance was in sharp contrast to other adipocytokines. RBP4 serum levels did not correlate with adiponectin, resistin or ghrelin serum concentrations, nor did adiponectin, resistin or ghrelin correlate with insulin resistance (data not shown).

### High RBP4 levels are a positive predictor of short-term survival at the ICU, but do not influence overall survival of ICU patients

**Figure 5:**
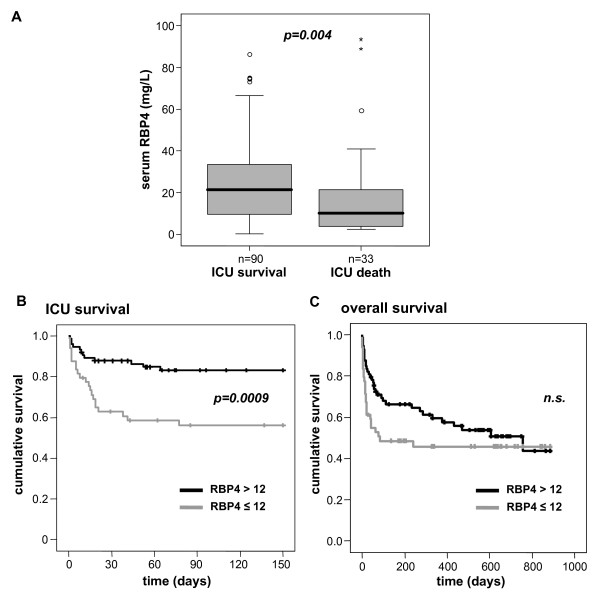
**Prognostic relevance of serum RBP4 in critically ill patients**. **(a) **Patients who die during the course of ICU treatment have significantly (*P *= 0.004) lower serum RBP4 levels on admittance to ICU than survivors. Boxplots are displayed, where the black bold line indicates the median per group, the box represents 50% of the values, and horizontal lines show minimum and maximum values of the calculated nonoutlier values; asterisks and open circles indicate outlier values. **(b) **Kaplan-Meier survival curves of ICU patients (*n *= 123) are displayed, showing that patients with high RBP4 levels (> 12 mg/L, black) have a decreased short-term mortality at ICU as compared to patients with low RBP4 (< 12 mg/L, gray). *P *value from Cox regression analysis is given. **(c) **Kaplan-Meier survival curves of ICU patients show no difference with respect to the long-term survival between patients with RBP4 levels (> 12 mg/L, black) and patients with low RBP4 (< 12 mg/L, gray). n.s., not significant.

We next analyzed whether serum RBP4 concentrations upon ICU admission may predict clinical outcome and mortality. Interestingly, patients who died during ICU hospitalization (33/123) displayed significantly lower serum RBP4 concentrations than the patients who were surviving (Figure 5a). The beneficial prognostic impact of high RBP4 levels on ICU survival was confirmed by Cox regression analysis (*P *= 0.031). By multivariate Cox regression analysis, the effect of RBP4 on ICU survival was independent of age, BMI, renal function (creatinine, GFR) and CRP, but indistinguishable from liver function. Two models were tested in multivariate Cox regression analyses, using ICU outcome (survival/death) as the endpoint. The first model included RBP4, age, BMI, creatinine, GFR (cystatin C-based) and CRP, and RBP4 was the only parameter that remained independently significant (*P *= 0.041) to predict ICU outcome in this combination. The second model combined RBP4 and markers of hepatic dysfunction (albumin, international normalized ratio, pseudocholinesterase). In this model, pseudocholinesterase remained the only independently significant (*P *< 0.001) parameter predicting ICU survival. If all parameters were combined, pseudocholinesterase was the dominant predictor for ICU outcome.

Using Kaplan-Meier survival curves with a cutoff for serum RBP4 concentrations of 12 mg/L (Figure 5b), high RBP4 levels were a significant positive prognostic marker for ICU survival (log-rank test, 10.96; *P *= 0.0009). On the other hand, serum RBP4 levels did not predict the overall survival of the ICU patients in the approximately 3-year follow-up (Figure 5c), indicating that the prognostic relevance and potentially also the pathogenic involvement of RBP4 is restricted to the acute illness.

## Discussion

RBP4, the circulating transporter for vitamin A, has recently been recognized as an important mediator of insulin resistance in mice and humans, but its potential role in sepsis or critical illness is presently a matter being researched. A recent single-center study reported low circulating levels of RBP4 in patients with acute critical illness of respiratory etiology compared to healthy controls. In the cohort of septic patients with identified pulmonary focus, lower levels of RBP4 were found than in nonseptic patients [15]. The authors hypothesized that an acute decrease of RBP4 concentrations could be explained by reduced synthesis or increased removal by extravasation due to capillary leakage or increased metabolic clearance [15].

Our study shows that reduced serum concentrations of RBP4 are a general response in critically ill patients, independent of the origin of the critical illness (sepsis or nonsepsis). The source of circulating RBP4 in critically ill patients appears to be primarily a combination of two factors: hepatic synthesis and renal clearance.

It is well established that the liver is the main source of RBP4 in humans [11,12], although adipocyte RBP4 secretion has been linked to obesity, insulin resistance and type 2 diabetes [12,13,25]. Consequently, patients with chronic liver diseases and liver cirrhosis have significantly decreased RBP4 serum concentrations, and serum RBP4 is directly linked to liver function in these patients [18,26,27]. Interestingly, we also observed a close correlation between the hepatic biosynthetic function and serum RBP4 in ICU patients, suggesting that circulating serum RBP4 is a direct consequence of hepatic synthesis comparable to the immediately increased hepatic synthesis of many acute phase proteins in critical illness. Furthermore, the lack of correlation between RBP4 and other adipokines indicates that the adipose tissue does not extensively contribute to serum RBP4 in patients with critical illness.

In addition, it is well established that serum RBP4 is increased in patients with chronic renal failure, making renal clearance a relevant confounding factor for serum RBP4 concentrations in patients with diabetes or renal disease [28,29]. As acute renal failure, mainly of prerenal origin during hemodynamic deterioration, is a classical feature of critically ill patients, our study demonstrated a direct correlation between increasing serum RBP4 and decreasing renal function in ICU patients. In multivariate analysis, hepatic and renal biomarkers independently correlated with serum RBP4 (data not shown), indicating that both mechanisms occur in parallel in critically ill patients regulating serum RBP4 levels. However, overall RBP4 serum levels remained significantly lower than in healthy controls.

Our results further indicate that the elevated serum RBP4 might be involved in the development of insulin resistance in ICU patients. Hyperglycemia and insulin resistance are well-known features of critical illness and affect the mortality rate, especially in septic patients [1,30,31]. Prior studies have confirmed excessive endogenous insulin secretion during sepsis, which represented an important pathogenetic and prognostic factor in ICU patients [30,32]. We show here that the circulating RBP4 level in ICU patients is not the result of preexisting diabetes or glucose intolerance (as reflected by HbA1c or plasma glucose). However, we did see a correlation with current endogenous insulin secretion, reflected by elevated C-peptide levels, and insulin resistance as calculated by the HOMA index, indicating that circulating RBP4 levels in ICU patients may contribute to the insulin-resistant state frequently observed in critically ill patients. Further studies in appropriate animal models of experimental sepsis are needed to clarify the potential pathogenetic role and metabolic consequences of RBP4 in critical illness.

The reduction of serum RBP4 in critically ill patients compared to controls was also of direct prognostic relevance. Our data revealed an association between low RBP4 and higher short-term mortality on ICU, whereas long-term survival was not affected. The underlying pathomechanisms are currently unclear. One explanation could be that this is a simple epiphenomenon as high(er) RBP4 levels may indicate preserved liver function; along this line, high "classical" biomarkers of liver function are also associated with a beneficial outcome (data not shown), and the predictive value of serum RBP4 for ICU survival was statistically indistinguishable from liver function in multivariate Cox regression analysis.

On the other hand, other explanations should be considered. Interestingly, serum RBP4 levels were negatively correlated with markers of inflammation. This raises the possibility that RBP4 could be an important component of the so-called anti-acute phase proteins [24], at least in patients with sepsis. In line with this possibility, decreasing serum RBP4 levels have been reported after acute surgery [33]. Functionally, elevated circulating RBP4 increased blood glucose in mouse models by inhibiting insulin signaling in skeletal muscle and upregulating gluconeogenesis through increased expression of the gluconeogenic enzyme phosphoenolpyruvate carboxykinase (PEPCK) in the liver [12]. The concomitant reduction in muscular glucose uptake and increase in hepatic glucose output could possibly favor an adequate stress response in the initial phase of critical illness and sepsis, which would explain the positive association between relatively high RBP4 and recovery from acute illness.

## Conclusions

Our study has demonstrated reduced serum RBP4 concentrations in critically ill patients prior to intensive care treatment measures. RBP4 serum levels at ICU admission appeared independent of underlying disease etiology, but were strongly associated with hepatic and renal function. The correlation with insulin resistance and the relation with the acute mortality collectively suggest that RBP4 may have implications for the pathogenesis of critical illness and could serve a novel biomarker for ICU patients. Future studies should consider including RBP4 as a factor for multivariate analyses and aim to unravel the kinetics of RBP4 levels in the time course of critical illness.

## Key messages

• Retinol-binding protein 4 (RBP4) has been suggested to contribute to insulin resistance, a common characteristic of critically ill patients with implications for adverse outcome.

• RBP4 serum levels are significantly reduced in critically ill patients as compared to healthy controls.

• RBP4 serum levels at ICU admission prior to intensive care measures appeared independent of underlying disease etiology.

• Serum RBP4 concentrations are closely related to liver and kidney function and correlate with endogenous insulin secretion and resistance.

• The negative association of serum RBP4 with inflammatory markers suggests a potential role in the anti-acute phase response in critical illness.

• Low RBP4 levels are an adverse predictor of short-term mortality at the ICU.

## Abbreviations

APACHE II: Acute Physiology and Chronic Health Evaluation; BMI: body mass index; CRP: C-reactive protein; ELISA: enzyme-linked immunosorbent assay; GFR: glomerular filtration rate; HOMA-IR: homeostasis model assessment index of insulin resistance; ICU: Intensive Care Unit; *P*: *P *value; PCHE: pseudocholinesterase; *r*: correlation coefficient; RBP4: retinol binding protein 4; SAPS: Simplified Acute Physiology Score; SIRS: systemic inflammatory response syndrome.

## Competing interests

The authors declare that they have no competing interests.

## Authors' contributions

AK, FT and CT designed the study, analyzed data and wrote the manuscript, RW performed RBP4 and adipokine measurements, and ES, HZ, HD and SV collected data and assisted in patient recruitment.
